# Modern Technologies in *Demodex* Blepharitis Diagnosis and Therapy (Review)

**DOI:** 10.17691/stm2025.17.3.06

**Published:** 2025-06-30

**Authors:** G.S. Igonin, S.N. Svetozarskiy, I.G. Smetankin

**Affiliations:** PhD Student, Department of Eye Diseases; Privolzhsky Research Medical University, 10/1 Minin and Pozharsky Square, Nizhny Novgorod, 603005, Russia; Ophthalmologist; Privolzhsky District Medical Center of Federal Medico-Biologic Agency of Russia, 2 Nizhnevolzhskaya naberezhnaya St., Nizhny Novgorod, 603001, Russia; Teaching Assistant, Department of Eye Diseases; Privolzhsky Research Medical University, 10/1 Minin and Pozharsky Square, Nizhny Novgorod, 603005, Russia; Associate Professor, Department of Eye Diseases; Privolzhsky Research Medical University, 10/1 Minin and Pozharsky Square, Nizhny Novgorod, 603005, Russia

**Keywords:** *Demodex* blepharitis, *Demodex*, demodecosis, blepharitis diagnostics, optical coherence tomography, confocal microscopy, machine learning

## Abstract

Blepharitis associated with *Demodex* infestation is a widespread condition, its complications include eyelid margin deformities, corneal erosions, and ulcers.

The review considers the epidemiological and pathogenetic aspects, along with new trends in *Demodex* blepharitis diagnosis and treatment, and represents the comparative characteristics of current diagnostic modalities, including traditional light microscopy, lateral eyelash retraction and rotation, as well as intravital imaging technologies, such as confocal microscopy and optical coherence tomography. Improving imaging techniques using machine learning was found to enable to improve early diagnosis availability and provide early initiation of etiotropic therapy. The review analyzes the preparations for conservative treatment of *Demodex* blepharitis,representing them with regard to the specificity of pharmacological effects and systemic safety, special attention being given to the problems of toxicity and shelf-life expectancy of drugs. Combination drugs and different laser exposure effects on *Demodex* mites and eyelid margin structures were stated to be prospective and understudied treatment approaches. We demonstrated the heterogeneity of approaches to efficacy assessment of diagnostic and therapeutic methods that makes actual the necessity of developing a standardized scale of *Demodex* blepharitis severity; the scale reflecting both clinical characteristics and instrumental findings. The authors concluded that the development of noninvasive imaging techniques and the shortest and safest therapeutic algorithms would enable to switch over to a whole new level of therapy efficacy for patients with *Demodex* blepharitis.

## Introduction

Blepharites are referred to an inflammatory disease of eyelid margins accompanied by itching, redness, exfoliation, and the formation of specific deposits. Both children and adults are susceptible to the disease [1]. By etiopathogenesis, blepharites have multifactorial pathology, in their development the key role is played by infectious agents, demodectic invasion, atopy, and seborrhea [2]. By the process location, there are anterior, posterior, and mixed blepharites [2, 3]. Anterior blepharitis is characterized by the inflammation of ciliary eyelid margin structures; it is most frequently caused by *Demodex* mites, staphylococcal infection, and seborrhea [3, 4]. In posterior blepharitis, the eyelid part, which is in contact with cornea and bulbar conjunctiva, is affected, primarily due to demodectic invasion. A mite is found on epilated eyelashes in 29% of examined patients aged from 0 to 25 years, in 53% — at the age of 26–50 years, in 67% of patients over 50 years [5–8].

The problem relevance of the early diagnosis and etiotropic treatment of demodectic etiology is related to its potential complications, including eyelid margin deformities, dry eye syndrome, corneal erosions, and ulcers [9]. Of particular interest are the feasibility study of current diagnostic technologies, including intravital imaging techniques and machine learning (artificial intelligence) for noninvasive assessment of eyelid margin condition, along with the evidence base analysis regarding the existing schemes of complex therapeutic and device treatment of *Demodex* blepharitis [10, 11].

## Literature search strategy

There were searched the articles in databases PubMed, eLLIBRARY.RU and Google Scholar published within the period 1970–2023 by the following key words: blepharitis, *Demodex* blepharitis, treatment, therapy, pharmacotherapy. Among 305 literature sources found, 101 scientific articles were recognized relevant for the review by the criteria of thematic reference.

## Biological background

*Demodex* (Gr. *demos* — wax or fat, *dex* — insect) — a microscopic parasite belonging to Arachnida class, acarine order. It is one of the most common parasites in the human body. Jacob Henle was the first to describe *Demodex* mite in 1841; later Carl Gustav Theodor Simon classified it as a human mite, *Demodex*. Currently, there are 21 *Demodex* mite types [12, 13]. Among the discovered species there are only two ones described on a human body: *Demodex folliculorum longus* (*D. folliculorum*) and *Demodex folliculorum brevis* (*D. brevis*). The most active accumulation of mites is primarily found in the areas of increased sebum production, namely, in the facial area and the external auditory canal region.

The question of whether the mite is commensal, i.e., symbiote doing no harm, is still debatable. At present, it is commonly believed that the physiologically permissible number of mites is less than 5 mite units per 1 cm^2^ in skin disorders and not more than 2 units in eyelash impairments [8].

## Epidemiology

*Demodex* mite prevalence in world population is 41–70% [14]. Moreover, demodicosis prevalence increases with age, reaching 67–100% in the population over 50 years [5, 14]. Impaired secretion-producing and secretion-excreting functions of the meibomian glands, impaired dermal and epidermal integrity, exposure to sunlight, alcohol, smoking, stress, hot drinks, spicy food, and sudden temperature change can be risk factors [9, 15]. The presence of immunocompromising diseases such as HIV infection and lymphproliferative diseases, as well as regular taking of steroids, predispose to *Demodex* invasion [16].

According to some estimates, demodectic invasion in its relatively imperceptible course can cause 29–74% of cases of chronic blepharitis that accounts for the significant proportion of patients followed by an ophthalmologist [17]. Dermatologic diseases can be associated with *Demodex* mite on skin: particularly, the risk of *Demodex* blepharitis increases by 7–8 times in patients with acne rosacea [18]. Demodectic invasion more intensively develops on eyelid margin structures, less accessible for thorough hygiene compared with projecting facial parts such as the nose, eyebrows, cheekbones, and cheeks [18].

## *Demodex* mite anatomy and physiology

*Demodex* mite is colorless, hairless, has spindleshaped, cylindrical body with rings and four short limbs on either side of the anterior third of the body (*podosome*). The body build enables mites to move at a speed of 8–16 cm/h. The lower two thirds of the body (*opisthosoma*) are elongated and tapering off; moreover, in *D. folliculorum opisthosoma* it is longer than in *D. brevis*. The mite anatomy includes the chitin exoskeleton, genital opening on the back side, and the digestive system, which lacks the anal orifice [18].

After fertilization a female mite moves into the hair follicle or the sebaceous gland and lays about 20 eggs, 50–60 μm in size, and they are at egg development stage for 60 h. Then the eggs change into the larval stage (36 h), followed by protonymph (72 h) and deutonymph (60 h), after that they take on a shape of an adult mite, which returns into the follicular opening [18–22].

*Demodex* mite depends on a host and survives *ex vivo* for no more than several days. *D. folliculorum* occurs more frequently as large accumulations around the eyelashes, *D. brevis* is widely spread throughout the body. *D. folliculorum* eats follicular epithelial cells using a pair of piercing mouth parts called chelicerae, and *D. brevis* — the sebaceous gland epithelium in the same way.

Microscopy of eyelashes and DNA analysis of mites showed that in closely related people suffering from *Demodex* blepharitis the similarity between the mites is higher. It is the evidence of non-percutaneous channel of infection [18]. The genes related to energetic balance and glycolysis regulation, allergen movement and encoding, detoxication, and stress reaction have higher expression in *D. folliculorum* compared with other species of mites [23]. In the clinical context it indicates its predominant role in developing type 1 allergic reaction with manifestations in the form of erythema and itching [23]. Aspartate protease, the synthesis of which is encoded by *D. brevis* mite genes, is able to lyse the host skin and blood serum molecules; it helps the parasite to penetrate into the host skin.

## Pathogenesis

*Demodex* mite action as a pathological agent was considered in the study from a variety of perspectives. Particular attention was given to its direct damaging action on human tissues, the role in transmitting pathogenic bacteria, and the ability to cause hypersensitivity reactions.

### Direct damaging action of Demodex

The number of *D. folliculorum* increases towards the eyelash root, where the parasite consumes the epithelial cells of the hair follicle, damaging its structure resulting in eyelash irregular growth [9, 24, 25]. Hyperkeratinization around the eyelash base can develop in response that visually is determined as dandruff or cylindrically- shaped deposits. Moreover, *D. brevis* is able to mechanically block the meibomian gland openings, the dysfunction of the glands leading to the lacrimal lipid layer deficiency. *D. brevis* is found in the center of meibomian granulomas surrounded by epithelioid cells, histiocytes, fibroblasts, lymphocytes, and plasmacytes [26]. Accordingly, *Demodex* mites can cause recurrent chalazion [9].

### Demodex as a bacterial transmitter

On its surface *Demodex* can carry bacteria including streptococci and staphylococci. Bacterial generation inside the mite, particularly *Bacillus oleronius*, was found to stimulate the proliferation of mononuclear cells of peripheral blood in patients with rosacea [27]. After mite death the bacterial antigen level was revealed to increase manyfold. When a mite is destroyed, the substrate for bacterial colony growth forms; it also contributes to the progression of inflammatory reaction cascades in the host body [9]. Some researchers suppose that the toxins produced by certain *Staphylococcus aureus* or *Staphylococcus epidermidis* strains absorbed on the mite surface can cause the inflammation [9]. Increased intensity of cell immunity to *S. aureus* was found in 40% of patients with blepharitis [28, 29].

### Hypersensitivity reaction

Delayed hypersensitivity can be caused by *Demodex* mite proteins in invasion in the eyelash follicle that is proved by the presence of Th cells in the inflammation area. The increased amount of macrophages and Langerhans cells was observed only in patients with positive *D. folliculorum* [9].

## Clinical presentation

Typical symptoms of demodectic invasion are itching in eyelids, the obstruction of meibomian ducts, chronic conjunctivitis signs, yellowish cylindrically-shaped deposits around the eyelashes, and trichiasis [8, 9]. Trichiasis resulted from the damaged eyelash follicle can injure the corneal epithelium, causing punctate erosions followed by corneal ulceration and pannus formation in severe cases [9]. In obstruction of excretory ducts of the meibomian glands by *Demodex* mites the secretion of lacrimal lipid component is impaired, resulting oftimes in accelerated tear evaporation and rupture, aggravating the corneal condition [9].

Delay in the disease diagnosis and treatment can lead to blepharoconjunctivitis, which is not arrested by standard anti-inflammatory therapy [9]. Itching in the eyelid area in *D. folliculorum* invasion, palpebral edema, and discomfort in the eyes are ones of the most important symptoms in the clinical presentation of the disease. The existing positive correlation between the increased number of *Demodex* mites and itching intensity increase is most likely to be due to the overexpression of genes encoding the allergens responsible for type I allergic reaction [18].

**Figure 1.**
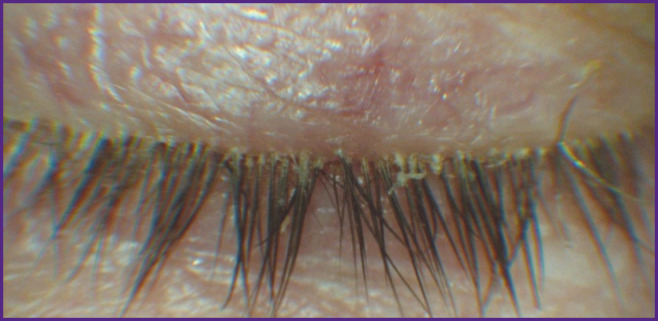
Photo of the eyelid margins of *Demodex* blepharitis patient At the eyelash roots there are yellowish cylindrical deposits consisting of *Demodex* mites and their waste products (the photo is from the authors’ photograph library)

On examination using a slit lamp, in most cases yellowish cylindrically-shaped deposits can be found around the eyelashes (Figure 1); the deposits representing the accumulation of *Demodex* mites and their waste products in the form of creatinine and lipids; their presence is considered to be highly specific for *Demodex* mites [30–32].

*D. brevis* able to penetrate deeply into the meibomian gland is supposed to be the risk factor of recurrent chalazion, and it should be taken into consideration if a patient complains of no effect of the administered conservative treatment and surgical therapy [33].

## Diagnosis

### Microscopy

**Figure 2.**
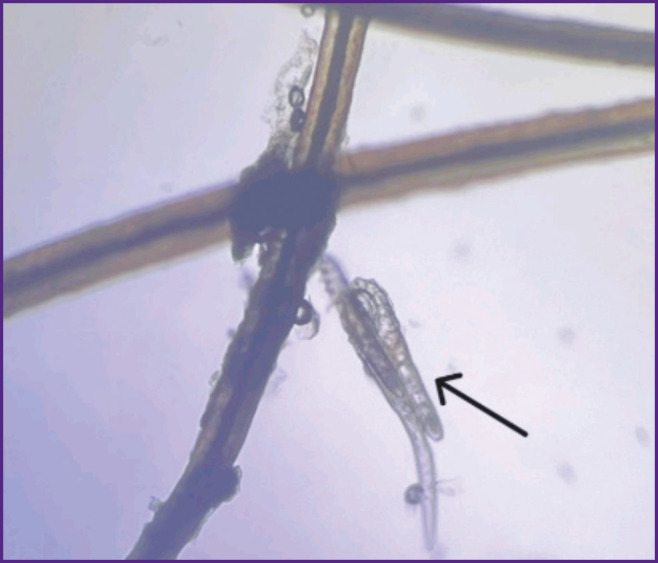
Light microscopy of *Demodex* blepharitis patient’s eyelashes The arrow indicates the adult *Demodex* mite (the photo is from the authors’ photograph library)

Traditional diagnostic methods of blepharitis associated with *Demodex* mite consist in the microscopic examination of eyelashes to assess the mite amount, morphology, and mobility [34]. The sampling is performed on a patient’s examination using a slit lamp; the eyelashes with cylindrical deposits are preferable to choose since they are found to have more mites [35]. The eyelash should be held close to the base, isolated, and placed on the object slide for microscopy (Figure 2). Additionally, it is possible to modify the method using fluorescein to stain chitin covering a mite for better imaging [32]. In mass invasion, when eyelash follicles are overfull, some mites accumulate on the ciliary margin near the follicle, however, due to their photosensitivity it rarely occurs [18].

Microscopy is the most common diagnostic method due to non-high requirements to a specialist to perform the procedure. The major drawback of light microscopy is an invasive character of monitoring, when repeated epilation is necessary that results in patient’s discomfort, since the procedure is painful. Other method limitations are a mechanical action in sampling and incomplete removal of mites from the follicular foramen. The addition of fixing agents for light microscopy can influence the mite morphological structure and its mobility, as well as the technique sensitivity [34]. The difficulty in diagnosis both *in vivo* and *ex vivo* is to differentiate the mite intruded in cylindrical deposits on the eyelashes. *Demodex* blepharitis diagnosis requires the light microscope with magnification 100–200, fixing agents, pipettes, a microscopic slide, and a cover glass, etc., all this mentioned imposes restrictions on implementing the method in common clinical ophthalmological practice.

### Eyelash rotation and lateral retraction

Rotation motions and lateral retraction of eyelashes are used as a rapid diagnostic test, it consisting in swinging an eyelash clockwise or counterclockwise in order to remove mites from the follicle. Further, *Demodex* mites can be calculated using a slit lamp with high magnification [36].

### Confocal microscopy

Confocal microscopy is a type of light microscopy. The method provides intravital imaging of the eye structures and its adnexa with very high resolution, due to which the method is widely used to diagnose corneal and conjunctival diseases [37, 38]. The use of confocal microscopy in blepharitis diagnosis is restricted. It is related to the anatomical position, mobility, and heterogeneous reflective capacity of the tightly fitting heterogeneous histological structures of the eyelid margin [37].

Considerable experience in confocal microscopy application has been gained in dermatological practice, where the technology enables to image *Demodex* mite as rosacea agent in the skin surface layers. The study by Sattler [38] et al. showed confocal microscopy to provide both qualitative and quantitative high-speed assessment of the scanned area.

The ophthalmological studies using confocal microscopy succeeded in revealing *D. folliculorum* inside the follicle, on the follicular bottom, near the meibomian gland, and between the eyelashes [39]. Dead *D. folliculorum* mites were found attached to the eyelash base. If the mite population was great, there were observed the inflammatory reaction signs [37]. *D. brevis* was determined on the follicular bottom or inside the meibomian gland. In case of the gland duct was obstructed by parasites, there was revealed epithelial proliferation. *Demodex* eggs were visualized with difficulty due to their small size. *Demodex* mite invasion was found in 60% of patients with dry eye syndrome and in 100% of patients with blepharitis [37].

### Optical coherence tomography (OCT)

OCT is an intravital technique for imaging the structure of optically heterogeneous media with high spatial resolution (5–10 μm). Light radiation in the infrared band enables to study skin structures at the depth of 750 μm. In dermatology, OCT is intensively applied to diagnose skin cancer, measure the tumor thickness in melanocytic lesions, and assess the epidermal changes followed local procedures [40–42]. Moreover, OCT is used as a noninvasive method to detect and quantitatively assess mite infestation in patients with *Demodex*-associated diseases. High resolution OCT in the *en face* mode managed to describe *Demodex* mite as an assembly of bright hyperreflective round points in groups of 3–5 mites per a hair follicle. The method sensitivity in diagnosis of *Demodex*-associated skin lesions, according to a pilot study on a group of 22 patients, approaches 100%, its specificity reaching 65% [43].

Currently, there are no studies devoted to OCT used to diagnose *Demodex* blepharitis. However, OCT is supposed to be a promising technique to study the nosology considering its sufficient penetrating power, noninvasive examination character, the rate of performing procedures, and the technology availability in modern ophthalmological clinics.

### Meibography

The method compared with the above mentioned ones is not a direct imaging technique of *Demodex* mites; however, it enables to judge the condition of the meibomian glands, the degree of their dysfunction and atrophy. In meibomian gland dysfunction, there is impaired secretion production, which hinders the tear evaporation, and the lacrimal film is necessary to level the corneal surface and create the regular optical medium. Mite infestation and waste products are supposed to cause the duct obstruction and trigger an inflammatory reaction; therefore, the assessment of the meibomian gland condition can be the indirect evidence of *Demodex* mite presence [6]. Meibography is based on intravital imaging of the meibomian glands in order to assess their morphology.

### Artificial intelligence

Owing to computation capacity development, advances in algorithms and architectures of machine learning, along with the availability of large data volumes, computer-assisted diagnostics of diseases has become one of the most intensively developed medical field worldwide [44, 45]. Artificial neuron networks abundantly used in recognizing images, speech, as well as the natural language processing are in the phase of being advanced into practical healthcare [10].

In ophthalmology, artificial intelligence is used to identify the ocular fundus photos, OCT scans and analysis of visual fields, and also to diagnose diabetic retinopathy and retinopathy of prematurity, glaucoma, macular edema, age-related macular degeneration [45]. The main objective of developing the technology is to improve the availability of medical care in terms of prevention, diagnostics, and treatment of ocular organs [10, 46]. There were developed the methods of recognizing keratoconus by photographs of the anterior eye segment based on deep machine learning with accuracy 97.6–99.3%, infectious keratitis with probability 90.7%, bacterial and fungal keratitis with specificity 76.5 and 100%, respectively [47, 48]. For these purposes, the use of artificial intelligence to diagnose *Demodex* blepharitis by the eyelid margin photos seems to be promising due to its simplicity and the non-invasiveness of obtaining primary data.

Machine learning application for recognizing confocal microscopic images demonstrates promising results. Developed neural network has enabled to classify patients by the presence of diabetic peripheral neuropathy by corneal nerve fiber length, sensitivity being 92% and specificity — 80% [49, 50]. Machine learning algorithms are also used for computer-aided evaluation of the meibomian gland height, width, tortuosity, and density in meibography [11]. In patients with *Demodex* blepharitis the meibomian gland atrophy is characterized by greater inhomogeneity than in patients with dystrophic changes of the meibomian glands without proved *Demodex* infestation. The changes in the meibomian gland height and density of the upper eyelid have more significant correlation with their dysfunction compared with the lower eyelid glands. It can be related to the fact that the upper eyelid margins are mostly exposed to mites, and the glands in upper eyelids are longer, it resulting in more pronounced atrophy [11].

Further implementation of artificial intelligence algorithms into the protocols of confocal microscopy and OCT has enabled to reduce the requirements to personnel training and objectify the technique making it more available. A multimodal study of the eyelid margins with automized analysis using neural networks is supposed to be a promising stage of developing the differential diagnostic methods of blepharitis [51].

### Efficacy comparison of the methods

Muntz et al. [34] compared the efficacy of different diagnostic methods: standard light microscopy, rotational technique, lateral eyelash retraction, and confocal microscopy. There were found no significant differences between the eyelash rotation and standard light microscopy of epilated eyelashes. Removing cylindric sheaths under light microscope was found to reveal mites twice as much than when using the rotation technique. Lateral eyelash retraction enabled to visualize most cigarshaped *Demodex* tails. Confocal microscopy exhibited low informativity of images, which made it difficult to differentiate *Demodex* mite that previously was detected by light microscopy.

Summing up the advantages and disadvantages of different methods, common diagnostic modalities can be ranged from the most specific and invasive light microscopy to less accurate method but non-contact, e.g., meibography (see the Table). Additionally, the comparison of traditional and modern approaches to the diagnostics of blepharitis caused by *Demodex* mite enables to determine the diagnostic signs of the disease:

long-term past history, the presence of confirmed *Demodex* blepharitis in close family;
chronic blepharitis, conjunctivitis, blepharoconjunctivitis, and recurrent chalazion, refractory to traditional treatment methods;
the presence of madarosis, trichiasis, refractory itching, eyelid redness, cylindrical sheaths on eyelashes;
detection of *Demodex* mites using imaging techniques at any life cycle stage.

**Table:** Comparative characteristics of diagnostic methods for *Demodex* blepharitis

Characteristics	Light microscopy	Eyelash rotation/lateral retraction in biomicroscopy	Confocal microscopy	Meibography
Requirements to researcher qualification	−	−	+	+
Invasiveness	+	−	−	−
Method availability	+	+	−	−
Identification of Demodex mites	+	+/−	+	−

N o t e: “+” — characteristic typical of the method; “−” — characteristics not typical of the method, “+/−” characteristic is not always provided when using the method.

## Therapy

*Demodex* blepharitis therapy aims at eliminating or significant reducing the number of *Demodex* mites. The requirement to therapeutic modalities is to achieve high efficacy in the shortest period and minimal adverse reactions.

### Medical therapy methods

Historically, *Demodex* blepharitis was treated by blepharal hygiene adding sulfur ointment, yellow salve or pilocarpine gel. Currently, neither sulfur ointment nor yellow salve is used, since their efficacy is lower. Pilocarpine in a gel form exhibits a sufficient antiparasitic effect, resulting in mite respiratory and mobility paralysis due to its action on the parasympathetic nervous system [7].

Among pharmaceuticals, special attention should be given to metronidazole and ivermectin. Metronidazole is antiprotozoal and antimicrobial, initially it was developed to treat infections caused by *Trichomonas vaginalis*, *Entamoeba histolytica*, and *Giardia lamblia*. Later, the pharmaceutical inhibiting the protein synthesis due to a microbial DNA destruction became widely used in the therapy of bacterial diseases [52–54]. The efficacy of metronidazole used locally in rosacea is explained its anti-inflammatory effect and the ability to reduce the density of follicles infected by *Demodex* mites [55]. Metronidazole monotherapy in *Demodex* blepharitis requires hard research. At present, the pharmaceutical has shown its high efficacy when used in combination with ivermectin [56].

Ivermectin is a broad-spectrum antiparasitic, its efficacy is due to its selective activity regarding glutamate-target chloride ion channels of the invertebrate peripheral nervous system [8]. Ivermectin binding to ion channels in the nerve and muscular cells, results in cell membrane permeability increase for chloride ions causing hyperpolarization followed by the parasite paralysis and death [57, 58]. Ivermectin hardly penetrates the human blood-brain barrier, since the mammals have ligand-gated ion channels, and owing these channels the risk the pharmaceutical has on the central nervous system is minimal [58]. The study of ivermectin efficacy in patients with *Demodex* blepharitis resistant to therapy demonstrated the reduction of *D. folliculorum* mite number, and improved characteristics of Schirmer tear test and the tear film rupture time according to 28-day therapy results [8]. The use of 1% ivermectin in the ointment form applied once a day for 2 months enabled to significantly decrease the intensity degree of cylindrical sheaths around the eyelashes from 3.37±0.70 to 0.1±0.3 scores, as well as the conjunctival redness — from 1.32±0.30 to 0.94±0.40 scores (when assessed in scores from 0 to 4, where 0 means no symptoms, 1 score — the presence of mild manifestations, 3 scores — mild disease, 4 scores — marked manifestations). Ocular surface disease index (OSDI) decreased from 58.74±17.90 to 17.10±10.50 scores [59]. In ivermectin monotherapy, as early as during the first therapy week the average number of *Demodex* mites reduced; however, on week 3 their number started increasing [58]. 21.7% of patients with ivermectin monotherapy had no clinical improvement, and 33.3% patients showed evident improvement, complete remission started in 45% of patients [58].

The use of combined therapy (metronidazole and ivermectin) resulted in significant *Demodex* infestation suppression throughout the observation period. So, the complete remission was recorded in 71.6% of patients, evident clinical improvement — in 26.7%. Clinical improvement was not found in 1.7% of patients [58].

The use of the combined gel containing 0.1% ivermectin and 1% metronidazole led to the complete eradication of *Demodex* mites in 96.6% of the subjects under study on day 30 of the observation [56].

An improved clinical presentation in the subgroups given the combined therapy can be explained by the fact that *Demodex* mite is able to cause an immune response resulting in inflammatory changes, while metronidazole acts as an anti-inflammatory component [58–62].

As a supplementary treatment method, in clinical practice there were the attempts to use tea tree oil. The latter is produced through steam distillation from *Melaleuca alternifolia* leaves. Since olden times, tea tree oil was used by Aborigines to treat wounds and skin infections [31, 63]. It has an antibacterial, antifungal, anti-inflammatory, and acaricidal effect [31, 64]. Tea tree oil contains a number of substances, and their main mechanism of action is inhibition of acetylcholinesterase resulting in the accumulation of acetylcholine in neural synapses, and causing neurotransmission impairment [65, 66]. The main active agent is terpinene-4-ol (T_4_O) [67–69].

*In vitro* studies showed *Demodex* mite to die within 90 min of 1% T_4_O action and within 40 min of 4% T_4_O action [33, 70]. In clinical practice the use of tea tree oil for a month resulted in the recovery of 7 from 9 patients with *Demodex* blepharitis [31, 71].

Tea tree oil exhibits high effectiveness, although the safety of its usage is still an open question, since it is found to have some adverse reactions. *D. brevis* mites are prone to site deeply in the meibomian gland that can require the longer drug exposition and result in Т_4_О cytotoxicity, which is caused by the dose- and time-dependent decrease in the survival of meibomian epithelial cells [33, 72]. The exposure to 1.0% T_4_O for 90 min results in the destruction of nearly all epithelial cells. If T_4_O content reduces to 0.01%, the cytotoxicity to the epithelial cells of the meibomian glands still preserves; however, such concentration keeps the ability to their differentiation [33, 73]. In personal hygiene products put up for sale Т_4_О concentration is higher, and the recommended scheme consists in using it twice a day for 6 weeks [74–77].

Tea tree oil is known to cause allergic contact dermatitis in 0.7% of patients, who underwent a patch test [78, 79]; forms secondary organic aerosols containing ultrafine particles, which can cause inflammation and an oxidative stress [80, 81]; contributes to developing the resistance to antibiotics in pathogens and saprophytic microorganisms if multiply used in the concentrations sublethal for bacteria (0.10–0.25%) [82, 83].

Tea tree oil has estrogen (0.025% tea tree oil) and antiandrogen (0.005% tea tree oil) activity [84]. Estrogens are the suppressors of sebaceous gland secretion and can promote the development of the meibomian gland dysfunction [78, 85–88]. Antiandrogenic substances can induce dry eye syndrome development, while androgens stimulate lipogenesis and prevent from the excess keratinization of the meibomian glands [89]. The meibomian gland is known to be the gland of holocrine type; therefore, its cells need continuous differentiation. However, when the substances with Т_4_О concentration over 0.01% are used, the process becomes impossible [33].

### Pulsed laser therapy

For demodecosis treatment, it is possible to use an impulse laser, wavelength 585 nm, which has a minimal effect on the surrounding tissues; however, the mechanism of action of the pulsed therapy on *Demodex* mite has not been fully investigated [90]. The skin absorption of a broad spectrum of beams from visible light to infrared light is supposed to result in heat elimination [91]. Laser pulsed therapy can influence the local immunity, the suppression of which enables *Demodex* mite to persist in skin layers. Indirectly, pulsed therapy is able to have an effect on mites regulating the level of transforming growth factor beta and the immunological mechanisms related to it [92]. *In vitro* experiments showed *Demodex* mites to live for a long time at temperature from 8 to 30°C, and the optimal temperature for their growth is within the range from 20 to 30°C [91, 93]. Temperatures below 0°C and above 37°C are not favorable to *Demodex* growth and development, 54°C is lethal temperature, and 58°C — the temperature necessary for effective mite eradication [91]. A group of researchers using real-time video-microscopy managed *in vitro* record *Demodex* mite death on the object slide when exposed to laser pulsed radiation; the mite death was characterized by motor activity stopping after five successive sessions of pulsed therapy when heating the object slide to 49°C [94]. It can be assumed that applying pulsed therapy succeeds in reaching the temperature, which promotes *Demodex* mite coagulation along with hair follicles preserved [92, 93].

*Demodex* mites have chromophores, which make the mite more sensitive to energy coming from the laser in pulsed therapy. Some researchers suggest that more spheric structures, such as *Demodex* mites, cannot accumulate and scatter the received energy. According to the performed studies, total eradication frequency was 55% after one-month pulsed therapy, and by 3 months it reached 100% [91, 95]. The comparative analysis of the pulsed therapy efficacy and 5% tea tree oil showed the mite eradication level in the first patient group to be 100%, and in the second group — 75% [96]. There is an assumption that pulsed therapy improves the meibomian gland secretion outflow due to telangiectasia reduction along the eyelid margin resulted from the effect on oxyhemoglobin, which like chromophore accumulates and converts the received energy into warm, due to which vessels dilate and the number of the molecules of inflammatory markers decrease [92, 95].

Laser with wavelength 577 nm was used to treat facial erythema, telangiectasia, nevi and rosacea [97]. The pilot study by Temiz et al. [97] showed that the laser exposure decreased *Demodex* density on week 4 of the therapy in 31 patients, and increased — in 3 patients, so it requires further studies. According to another group of researchers, there were found no significant differences between *Demodex* mite density before and after the laser treatment (the laser wavelength was 577 nm) [98].

In literature there was described the experience in applying Nd:YAG-laser with wavelength 1064 nm to treat facial erythema with telangiectasias of demodectic etiology [99]. According to the study findings, after two sessions with one-month interval the patients were found to have less symptom load, and the mite number reduced by 28.1%, the authors related it to the thermal effect on *Demodex* mites and their following destruction [99].

### Treatment modalities compared

When comparing the effectiveness of different therapy methods, it is necessary to take into consideration their exposure time. In one-month therapy the most pronounced effect was observed when ivermectin was systemically taken in combination with metronidazole; if the therapy lasted from 1 to 3 months, ivermectin used locally showed the best effect. Laser pulsed therapy demonstrates high eradication characteristics if treatment duration is over 3 months [100]. Exposure to intensive pulsed light, ivermectin, and tea tree oil used locally can provide nearly total elimination of *Demodex* mites [100, 101].

## Conclusion

Blepharitis of demodectic etiology is rather common condition, which, however, is not easy to diagnose. Accordingly, it is relevant to improve non-invasive technologies of intravital imaging using neuronal networks. It will enable to significantly increase the early diagnosis accuracy and provide the initiation of early etiotropic therapy. Conservative therapy should aim at improving the therapy efficiency and cocurrent decrease of the drug toxicity and their use time period. One of the ways to improve treatment modalities of *Demodex* blepharitis is to combine pharmaceuticals and laser techniques; however, such approach requires further research. Additionally, it is necessary to develop a standardized severity scale considering clinical characteristics, life quality and the findings of instrumental diagnostic methods. It will make it possible to objectively assess the disease course dynamics, and arrange the personalized management.
